# MicroRNA miR-92a-1 biogenesis and mRNA targeting is modulated by a tertiary contact within the miR-17∼92 microRNA cluster

**DOI:** 10.1093/nar/gku133

**Published:** 2014-02-11

**Authors:** Steven G. Chaulk, Zhizhong Xu, Mark J. N. Glover, Richard P. Fahlman

**Affiliations:** ^1^Department of Biochemistry, University of Alberta, Edmonton, Alberta T6G 2H7, Canada and ^2^Department of Oncology, University of Alberta, Edmonton, Alberta T6G 2H7, Canada

## Abstract

While functional mature microRNAs (miRNAs) are small ∼22 base oligonucleotides that target specific mRNAs, miRNAs are initially expressed as long transcripts (pri-miRNAs) that undergo sequential processing to yield the mature miRNAs. We have previously reported that the pri-miR-17∼92 cluster adopts a compact globular folded structure that internalizes a 3′ core domain resulting in reduced miRNA maturation and subsequent mRNA targeting. Using a site-specific photo-cross-linker we have identified a tertiary contact within the 3′ core domain of the pri-miRNA between a non-miRNA stem-loop and the pre-miR-19b hairpin. This tertiary contact is involved in the formation of the compact globular fold of the cluster while its disruption enhances miR-92a expression and mRNA targeting. We propose that this tertiary contact serves as a molecular scaffold to restrict expression of the proposed antiangiogenic miR-92a, allowing for the overall pro-angiogenic effect of miR-17∼92 expression.

## INTRODUCTION

**Figure 1.**
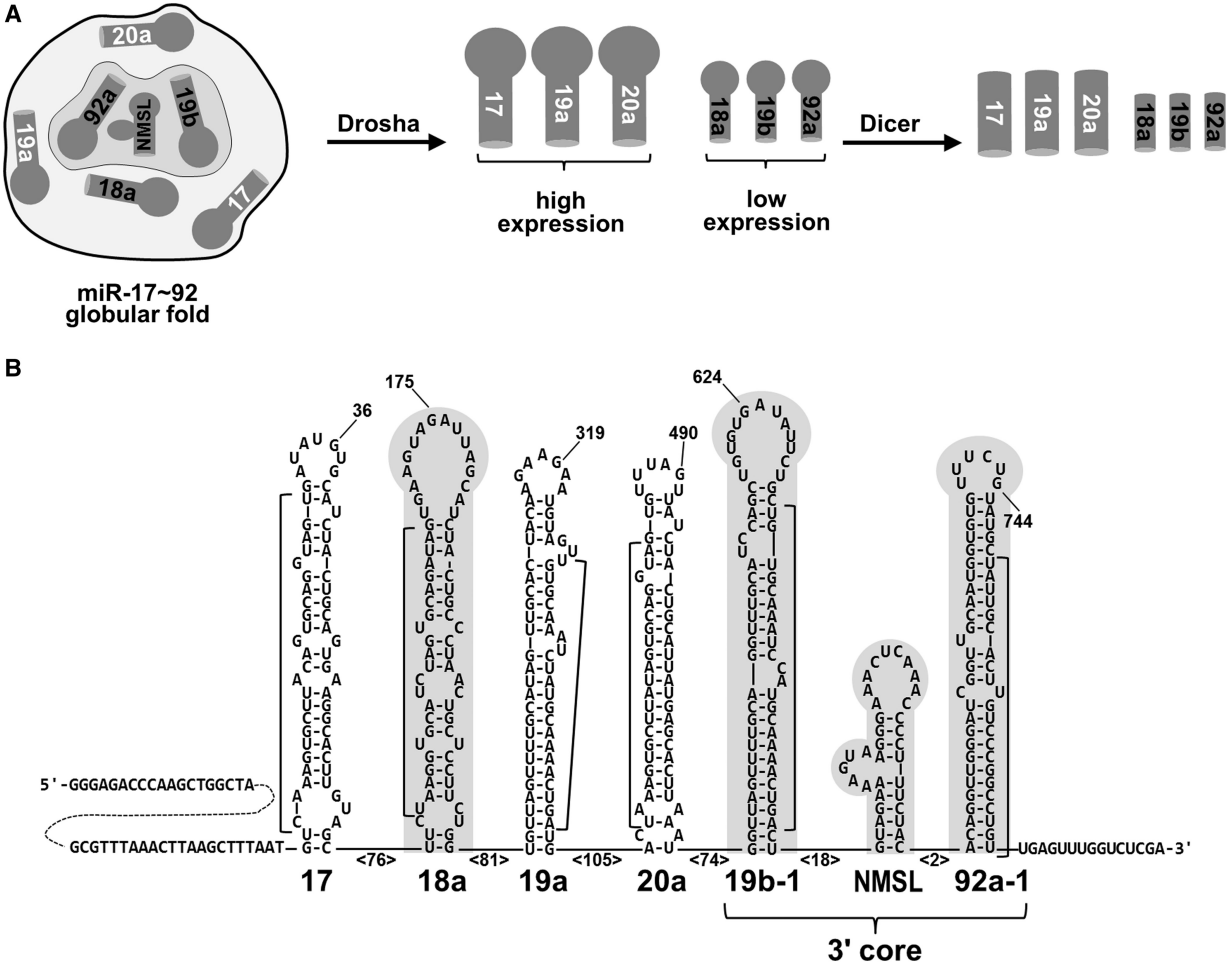
Structural regulation of miR-17∼92 miRNA processing. (**A**) The globular fold of the miR-17∼92 pri-miRNA internalizes miR-19b and miR-92a of the 3′ core domain as well as miR-18a. The internalized miRNAs have reduced expression relative to surface exposed miR-17, miR19a and miR20a due to suppressed Drosha processing. (**B**) Predicted secondary structure (mFold) of the miR17∼92 cluster. The sequence shown is that of the T7 RNA polymerase run-off transcript generated from Xho I–digested pcDNA 3.1 (+) vector. Select nucleotides are numbered for reference, the numbers in angle brackets indicate the number of intervening nucleotides. The internalized 3′ core domain and miR-18a hairpin are shaded gray and mature miRNA sequences are indicated with a bracket. Nucleotides 678–710 comprise the non-miRNA containing stem-loop (NMSL).

MicroRNAs (miRNAs) are small RNAs that regulate a wide variety of cellular processes from diverse organisms (1–6). Individual miRNAs are located within longer primary transcripts (pri-miRNA) containing one or more miRNAs. These long pri-miRNAs are processed into ∼70 nucleotide stem-loop RNAs by Drosha (Figure 1) (7). These ∼70-nt pre-miRNAs are then exported out of the nucleus to the cytoplasm by Exportin 5/Ran GTPase where they are processed by another type III ribonuclease, Dicer, into ∼22 nt double-stranded RNAs. (8). One strand of the miRNA molecule is then incorporated into an RNA-induced silencing complex, which is the complex that directs the RNAi mediated gene regulation by targeting a homologous mRNA (9).

OncomiRs are miRNA genes that function as oncogenes (10) and their expression may be central in the development of some cancers. One example of an oncomir is the *miR-17∼92* cluster (oncomiR-1), which contains six different miRNAs (Figure 1). The *mir-17∼92* cluster is located at *13q31.3*, a genomic locus that is amplified in several types of cancer and lymphomas (5,11). Further, overexpression of the miR-17∼92 cluster in a mouse B cell lymphoma model accelerates tumor development (12). The molecular mechanisms of these effects on cancer by miR-17∼92 have been demonstrated to involve both c-Myc and E2F transcription factors (13). The involvement of the miR-17∼92 cluster in cancer likely extends beyond its oncogenic activity as the genomic locus of miR-17∼92 is deleted in a subset of breast and cervical cancers (5). Experiments in mice have shown that expression of the miR-17∼92 cluster is essential, the genetic knockout of miR-17∼92 in mice results in the development of lymphomas and death soon after birth. Knockout of miR-17∼92 and one of its paraloguous clusters miR-106a or miR-106b, results in death at mid-gestation (14). Clearly, miR-17∼92 and its paralogues have a large impact on gene regulation, development and disease.

While the pri-miRNA secondary structural requirements for Drosha processing have been determined, the tertiary structural features of pri-miRNA clusters have been largely unexplored (15,16). The miRNA hairpin terminal loop, the ∼30 base-pair miRNA containing stem and the basal single-stranded tails have all been shown to be required for optimal Drosha processing. Just recently, an upstream UG sequence motif and a downstream SRp20 CNNC binding motif have been identified as primary sequence determinants for optimal Drosha processing (17). We recently reported that the pri-miR-17∼92 miRNA cluster adopts a compact globular structure (Figure 1) where the 5′ region of the cluster folds on a 3′ core domain containing miRNAs miR-19b and miR-92a (18). Independent of our initial investigation, another group has also reported that this cluster adopts a compact folded structure (19). In our previous report we demonstrated that the internalized miRNAs are processed less efficiently than those on the surface of the structure (Figure 1) (18). Disruption of the structure exposes the miRNAs within the 3′ core domain resulting in increased miR-92a expression in conjunction with increased repression of a validated miR-92a target, ITGA5 mRNA (18).

Here we report on the first confirmed tertiary structural element in the pri-miR-17∼92 cluster located within the 3′ core domain. By site-specific photo-cross-linking and verification by mutagenesis, we have identified a tertiary contact between hairpins within the 3′ core domain that is involved in the overall folding of the pri-miRNA structure.

## MATERIALS AND METHODS

### RNA preparation

miR-17∼92 was cloned from total HeLa RNA using reverse transcriptase-polymerase chain reaction (RT-PCR; miR-17∼92: First strand reverse oligo 5′-GCG CGC CTC GAG ACC AAA CTC AAC AGG CCG GGA CAA GTG CAA-3′ and forward primer 5′-GCG CGC GCA AGC TTT AAT GTC AAA GTG CTT ACA GT-3′) and inserted into a pcDNA 3.1(+) vector (Hind III and Xho I sites) to facilitate run-off transcription or transient transfection into HEK293T cells. 3′ core domain (nucleotides 518–781) T7 RNA polymerase templates were prepared by PCR, miR-17∼92 mutants and 3′ core domain mutants were prepared by PCR. The non-miRNA stem-loop (NMSL) was transcribed from a chemically synthesized T7 RNA polymerase template.

### RNA folding

RNAs were annealed using the following protocol: 90°C for 30 s, 70°C for 1 min, then slow cooled to 25°C over 20 min in 10 mM sodium cacodylate, pH 6.8, 10 mM NaCl, 10 mM MgCl_2_ and 0.1 mM EDTA. RNA folding was assayed using agarose gel electrophoresis [2.5% agarose, 2.5 mM MgCl_2_, tris (25 mM)-glycine (190 mM), pH 8.0], and RNAs were visualized by ethidium bromide staining.

### RNase T1 digestion

3′-end-labeling was performed with 5′-[^32^P]-pCp (Perkin Elmer, 3000 Ci/mmol) and RNA ligase1 (New England Biolabs). RNase T1 (Ambion Inc.) probing reactions were performed on 3′-[^32^P]-end-labeled magnesium annealed miR-17∼92 and 3′-[^32^P]-end-labeled magnesium annealed 3′ core domain. After the indicated times, the reactions were quenched by phenol/chloroform extraction and ethanol precipitation. Cleavage products were resolved by 6% (19:1) 8 M urea denaturing polyacrylamide gel electrophoresis (PAGE). Sites of cleavage were assigned by comparison with RNase T1 sequencing reactions, alkaline hydrolysis ladders and RNA size standards.

### RNase 1 and V1 digestion

5′-[^32^P]-end-labeling was performed with γ-[^32^P]-ATP (Perkin Elmer, 6000 Ci/mmol) and T4 polynucleotide kinase (New England Biolabs). 5′-end-labeled NMSL was incubated with either RNase 1 or V1 in the presence of yeast tRNA (0.05 µg/µl final concentration). Reactions were quenched by phenol/chloroform extraction and ethanol precipitation. Cleavage products were resolved by 20% (19:1) 8 M urea denaturing PAGE.

### 4-thio-uridine cross-linking

3′ core domain RNAs with site-specific 4-thio-uridine (4SU) incorporation at position 685 or 695 were prepared using a splinted ligation strategy:

GGC AGA UCU UAC UGC UAG CUG UAG AAC UCC AGC UUC GGC CUG UCG CCC AAU CAA ACU GUC CUG UUA CUG AAC ACU GUU CUA UGG UUA GUU UUG CAG GUU UGC AUC CAG CUG UGU GAU AUU CUG CUG UGC AAA UCC AUG CAA AAC UGA CUG UGG UAG UGA AAA GUC UGU AGA AAA G**(U685)**A AGG GAA AC**(U695)** CAA ACC CCU UUC UAC ACA GGU UGG GAU CGG UUG CAA UGC UGU GUU UCU GUA UGG UAU UGC ACU UGU CCC GGC CUG UUG AGU UUG GUA GG

RNA oligonucleotides with a single 4SU residue were purchased from Dharmacon: 4SU685, GUA GAA AAG (4SU)AA; 4SU695, AC(4SU) CAA ACC CCU UUC UAC ACA. The 5′ and 3′ portions for ligation to the 4-thio-U RNA were transcribed from PCR-generated T7 RNA polymerase templates. Both the 4-thio-U RNAs and the 3′ RNAs were phosphorylated with ATP and γ-[^32^P]-ATP, respectively. All three RNAs and a DNA splint [4SU685 DNA splint (5′-TGA GTT TCC CTT ACT TTT CTA CTT TTC TAC AG-3′), 4SU695 DNA splint (5′-GAT CCC AAC CTG TGT AGA AAG GGG TTT GAG TTT CCC TTA ct-3′)] were denatured at 95°C for 1 min followed by a 5-min 60°C and 5-min room temperature annealing. Ligation reactions were performed with T4 DNA ligase (3200 units, New England Biolabs). RNAs were annealed as described above and photolyzed for the indicated times on ice, with a 365 nM hand-held ultraviolet lamp (Entela) 2-cm away. Cross-links were mapped using cross-linked 10 pmol RNA as a template for Superscript III (Invitrogen) with 20 pmol 5′-end-labeled DNA probe (5′-TG AGT TTC CCT TAC TTT TCT ACT TTT CTA CAG-3′). RNA and probe were annealed and reverse transcription was performed at 55°C as per the manufacturer’s instructions. After reverse transcription, RNA was removed by RNase H treatment.

### Cellular miRNA processing

hsa-miR-1-1, miR-17∼92, NMSL-A/U and NMSLR-A/U constructs were inserted (KpnI and XhoI sites) into pcDNA 3.1 (+). HEK293T cells were cultured in Dulbecco's modified Eagle's medium (DMEM) media supplemented with 10% fetal bovine serum (FBS). Cells were transfected with 30 μg of total DNA (5 μg of miR-1-1 vector plus 25 μg of cluster vector) using the calcium phosphate method (20). Total RNA was prepared with 1 ml of Trizol (Invitrogen). Twenty micrograms of total RNA was resolved by 15% (19:1) denaturing PAGE and electrotransferred to GeneScreen Plus membrane (Perkin Elmer). Blots were probed at 42°C with 5′-[^32^P]-end-labeled DNA oligonucleotides [in UltraHyb Oligo buffer (Ambion)] complementary to the mature miRNA sequences. The human miR-1-1 pri-miRNA insert was generated by PCR: forward primer 5′-ATA CCG CTC GAG CTT CTG CCT TTC TGG ATC GTG T-3′; reverse primer 5′-ATA CCG CTC GAG CTG CTG ACA CAG GAA AGT GAC-3′ and was cloned into the XhoI site of pcDNA 3.1 (+). miRNA expression was quantified with ImageQuant (5.2) software. The sum of the amount of pre-miRNA and mature miRNA [corrected for lane loading (U6 probing) and transfection efficiency (miR-1-1 probing) and background endogenous expression] for each miRNA from each mutant cluster, was normalized to wild-type expression. Bar graphs were generated with Excel software.

### Measurement of ITGA5 mRNA levels

HEK293T cells were cultured in DMEM media supplemented with 10% FBS. Cells were transfected with 30 μg of DNA using the calcium phosphate method (20). Total RNA was prepared with 1 ml of Trizol (Invitrogen). Quantitative RT-PCR was done in two steps. cDNA was synthesized (Invitrogen SuperScript™ III Reverse Transcriptase, 30 min at 50°) using 5 µg of total RNA template; cDNA was then quantified by qPCR (Invitrogen SYBR® GreenER™ qPCR SuperMix Universal) using a Rotor-Gene RG-3000 (Corbett Research).

## RESULTS

### The NMSL and 3′ Core domain are internalized within the miR-17∼92 structure

**Figure 2.**
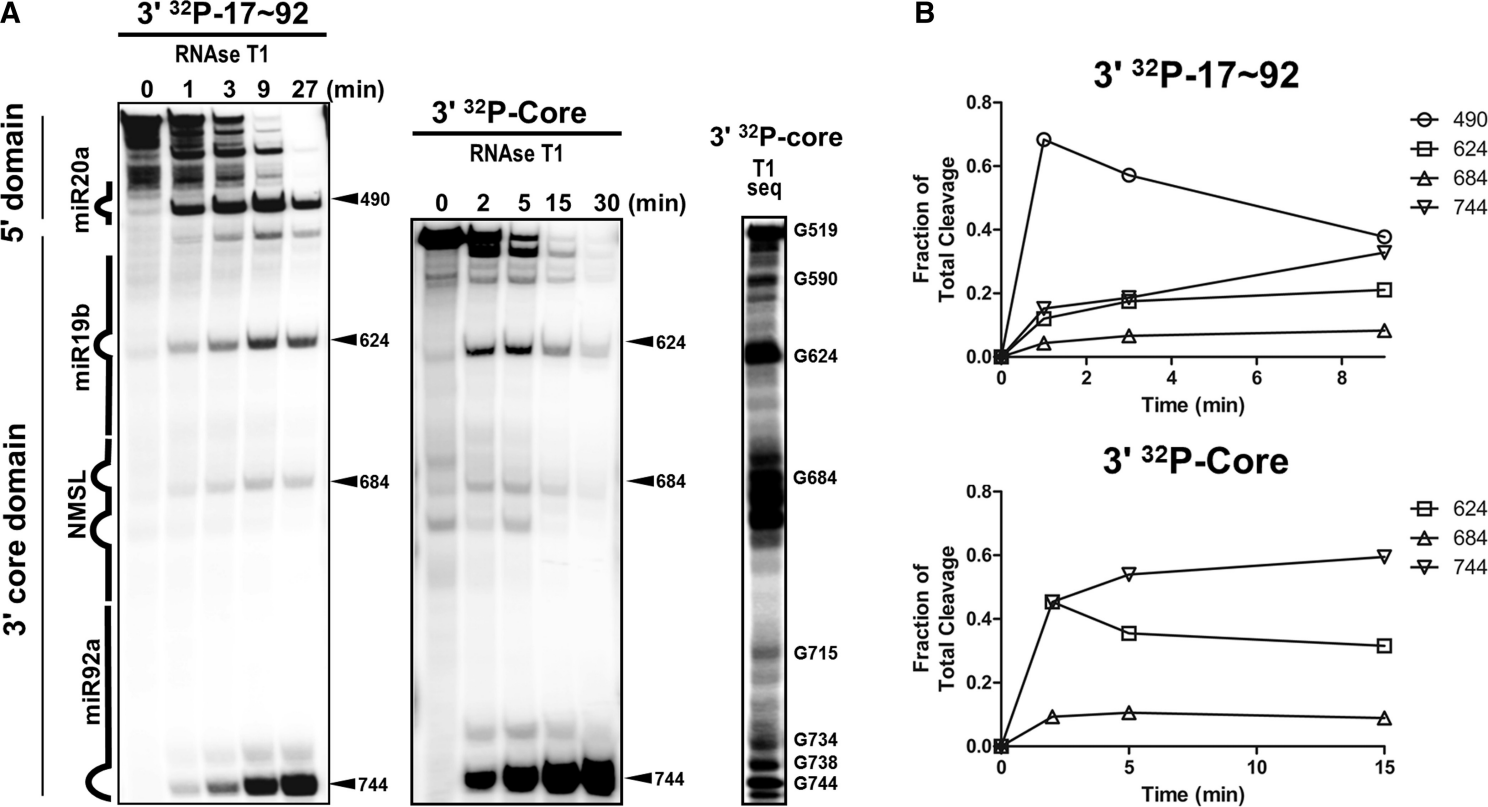
Structure probing of miR-17∼92 with an extended miR-17∼92 RNAse T1 digestion. (**A**) Time dependence of RNAse T1 digestion of 3′-end-labeled miR-17∼92 and 3′ core domain. Early major cleavages of miR-17∼92 occur outside the 3′ core domain. Over time, cleavage within the 3′ core domain becomes more prominent and resembles the cleavage pattern of the isolated 3′ core domain. Size standards and an RNAse T1 digestion under denaturing conditions (sequencing conditions) of 3′-[^32^P]-end-labeled 3′ core domain was used to assign cleavages. (**B**) Time dependence of RNAse T1 cleavage product generation. A plot of the amount of cleavage for each position (relative to total cleavage in the lane for that time point) versus time shows an early peak for cleavage outside the 3′ core domain. The NMSL is relatively protected throughout digestion of both miR-17∼92 and the 3′ core domain.

We previously reported (18) that the ∼800 nucleotide sequence containing the miR-17∼92 pri-miRNA adopts a globular structure that folds on an internalized ∼275-nucleotide 3′ core domain. Under foot-print probing conditions, the 3′ core domain is resistant to ribonuclease cleavage. We have now further analyzed the miR-17∼92 structure, in particular the 3′ core domain. RNAse T1 digestion time courses (non-footprint probing with complete digestion of the full-length input RNA) of the full-length pri-miR-17∼92 RNA domain or the 3′ core domain reveals differential time-dependent susceptibility of the miRNA hairpins and the NMSL (Figure 2A). Quantification of the RNAse T1 cleavage time courses are shown in Figure 2B. The earliest time point with the full-length pri-miRNA reveals major cleavages outside of the 3′ core domain, site 490, which is located within miR20a, that result in high molecular weight cleavage products as previously reported with RNA foot-printing experiments (18). As the cleavage reaction progresses and the majority of the full-length pri-miRNA is degraded, cleavages at sites 624, 684 and 744, which are all within the 3′ core domain, develop with the cleavage pattern resembling that observed with the isolated 3′ core domain. The temporal RNase T1 susceptibility of the 3′ core domain is consistent with a model where the 3′ domain is buried within the pri-miRNA structure. Within the 3′ core domain is the highly conserved NMSL that we previously identified as being involved in tertiary structure formation in the full-length cluster (18). In addition to being protected from both ribonuclease and hydroxyl radical probes within the full-length pri-miR-17∼92 sequence, the NMSL is also protected throughout the extended RNAse T1 digestion of the 3′ core domain (Figure 2), suggesting that the NMSL is internalized within the 3′ core domain. The significant cleavage observed in the NMSL when generating the RNAse T1 ladder under denaturing conditions (T1 seq, Figure 2A) additionally supports this hypothesis that this protection pattern is a result of RNA structure.

### Adenosine repeats within the NMSL

**Figure 3.**
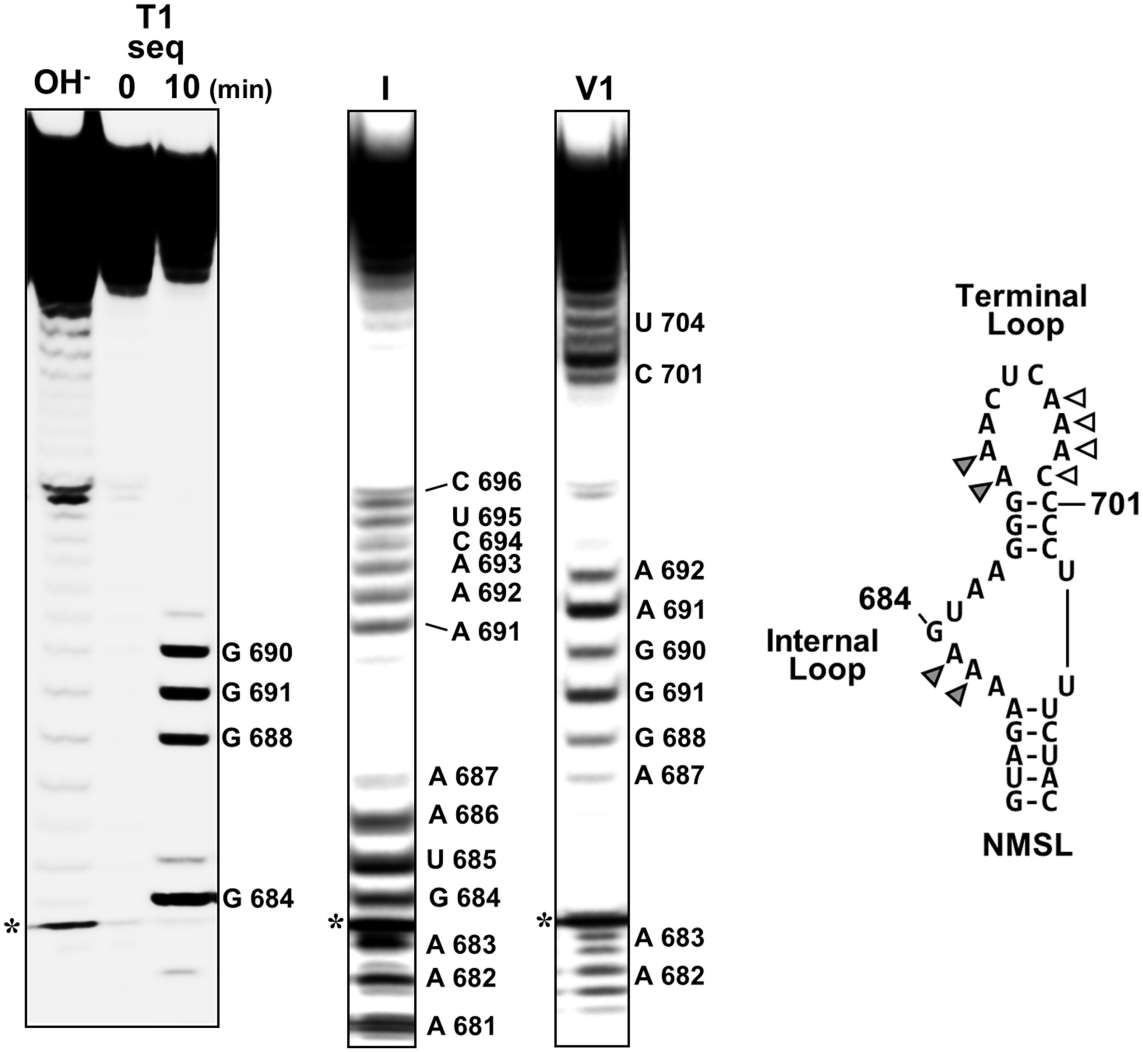
Structure probing of the NMSL. Denaturing PAGE analysis of RNAse 1 and RNAse V1 cleavage of 5′-[^32^P]-end-labeled NMSL. Cleavages were assigned using a base hydrolysis ladder and an RNAse T1 sequencing reaction. Adenosines that are cleaved by both RNase 1 and V1 are indicated by gray triangles. The white triangles indicate positions that are not cleaved by either RNase. The predicted Watson–Crick base pairs A681:U705 and A687:U704 could not be confirmed by ribonuclease sensitivity. The asterisk indicates a nonribonuclease degradation product. 3′ heterogeneity of the low molecular weight cleavage products for positions A 682 and A 683 are visible.

Considering the internalization of the NMSL and the high level of sequence conservation of the NMSL, we further focused on this predicted hairpin (18). As the NMSL is resistant to nuclease digestion within the core domain, structural probing on the isolated ∼40-nucleotide NMSL RNA was performed. RNase 1 (cleaves 3′ of single-stranded nucleotides) (21) and RNase V1 (cleaves 3′ of double-stranded, or single-stranded base-stacked nucleotides) (21–24) were used to determine the single- and double-stranded regions of the isolated NMSL sequence. Ribonuclease cleavages were visualized by denaturing gel electrophoresis (Figure 3). While most of the RNase 1 and RNase V1 cleavages were consistent with the base-pairing in the predicted secondary structure (25), adenosines 682 and 683 of the internal loop and 691 and 692 of the terminal loop are cleaved by both RNAse 1 and RNase V1. Though a high-resolution x-ray crystallographic or NMR analysis would be required to definitively confirm base stacking, the dual susceptibility to RNase 1 and RNAse V1, frequently seen in loops in other RNAs (26), is consistent with these adenosines being base-stacked while single stranded (26,27). Given the involvement of single-stranded adenosine repeats in RNA tertiary structure formation generally, and in miR-17∼92 in particular (19,28), along with the prevalence of single-stranded base-stacked adenosine platforms in adenosine-rich tertiary structure motifs (29), we next focused on whether these adenosines are involved in tertiary contacts in the miR-17∼92 structure.

### NMSL forms tertiary contacts with the miR-19b hairpin

**Figure 4.**
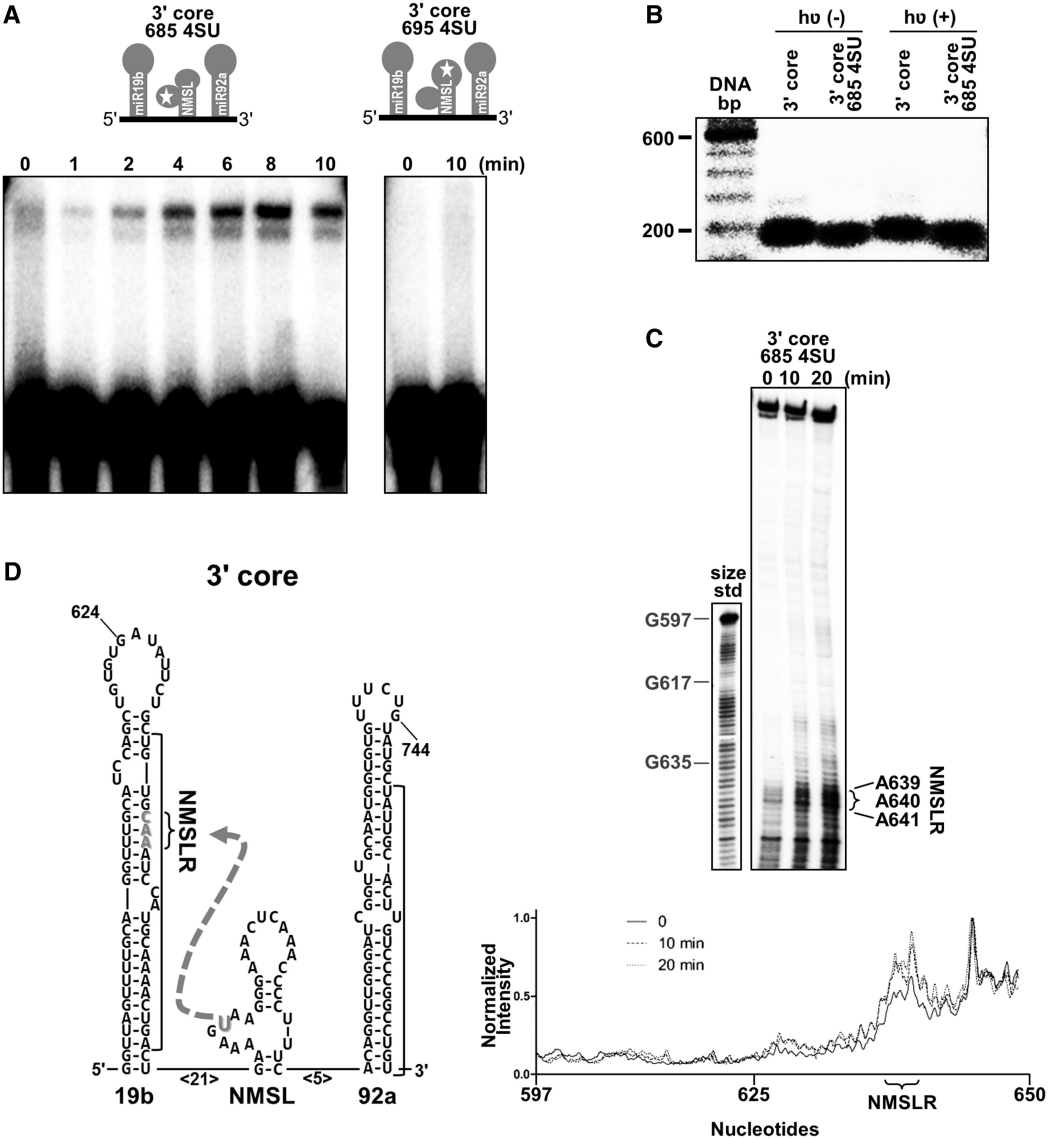
Tertiary contact between the NMSL and NMSLR. (**A**) Top, schematic of 4SU incorporation into the 3′ core domain at positions 685 (3′ core 685 4SU) or 695 (3′ core 695 4SU) for photo-cross-linking. Bottom panel, denaturing gel electrophoresis of 365 nm photolyzed 4SU-modified RNAs. Only the 3′ core 685 4SU RNA shows a cross-linked RNA of lower electrophoretic mobility. (**B**) Native gel electrophoresis (2.5 mM MgCl_2_ in the gel and running buffer) reveals that neither the incorporation of 4SU at position 685 of the NMSL, or photolysis, disrupts 3′ core domain structure. Wild-type 3′ core domain is 5′-[^32^P]-end-labeled, 3′ core 685 4SU has an internal ^32^P radio-label incorporated during splinted ligation. (**C**) Cross-link–dependent reverse transcription stops indicate the positions of the nucleotides cross-linked to the 4SU within the 3′ core. Positions of size standards corresponding to positions G597, G617 and G635 are indicated on a DNA ladder. Significant increases in band intensity are only observed for the NMSLR region. (**D**) Schematic of the hairpins within the 3′ core, with the cross-link between the NMSLR and the 4SU indicated by a dashed arrow.

Photo-cross-linking is a direct method to identify potential tertiary contacts (30–32) between different regions on an RNA. An RNA corresponding to the 3′ core domain was synthesized, using a splinted ligation strategy (33), to site-specifically incorporate a 4SU into the internal loop of the NMSL at nucleotide 685 (685 4SU) as schematically shown in Figure 4A. A second construct was also generated where the 4SU was incorporated in the terminal loop of the NMSL at position 695 (695 4SU) as shown schematically in Figure 4A. Photo-induced cross-links within an RNA sequence can generate a lariat structure that can be visualized by denaturing PAGE as a result of its decreased electrophoretic mobility (30,34). Photo-irradiation (365 nm light) of the 3′ core domain construct containing a 4SU incorporated in the internal loop exhibits a time-dependent formation of a molecular species with reduced electrophoretic mobility (Figure 4A). Similar photo-irradiation of the 3′ core domain construct with the 4SU incorporated into the NMSL terminal loop exhibited no formation of an observable cross-linked species when using similar irradiation times (Figure 4A). The 4SU incorporation in the NMSL internal loop does not disrupt the core domain structure, with or without photolysis, as assayed by native gel electrophoresis (Figure 4B). The observed lariat formation on irradiation of the 3′ core RNA with the 4SU in the internal loop indicates that this internal loop may be involved in a tertiary contact within the 3′ core domain.

A reverse transcription assay (35) was used to map the location of the 4SU photo-cross-link. RNA samples, where the 4SU was incorporated into the internal NMSL loop, were photo-cross-linked for 10 or 20 min and were then used as templates for reverse transcription reactions with a 5′-[^32^P]-end-labeled primer. The reverse transcription reactions were resolved by denaturing gel electrophoresis and visualized by autoradiography (Figure 4C). Significant increases in premature stopping in the reverse transcription reaction is observed at positions 639–641, which is located within the miR19b hairpin as shown schematically in Figure 4D. Thus, the 639–641 region of pre-miR-19b hairpin appears to act as a binding partner or receptor for the internal loop of the NMSL (Figure 4D). We have termed the 639–641 nucleotides the NMSL receptor (NMSLR). When the 4SU cross-linker is similarly incorporated into the full-length cluster, reverse transcription stops are also mapped to the NMSLR (Supplementary Figure S1).

**Figure 5.**
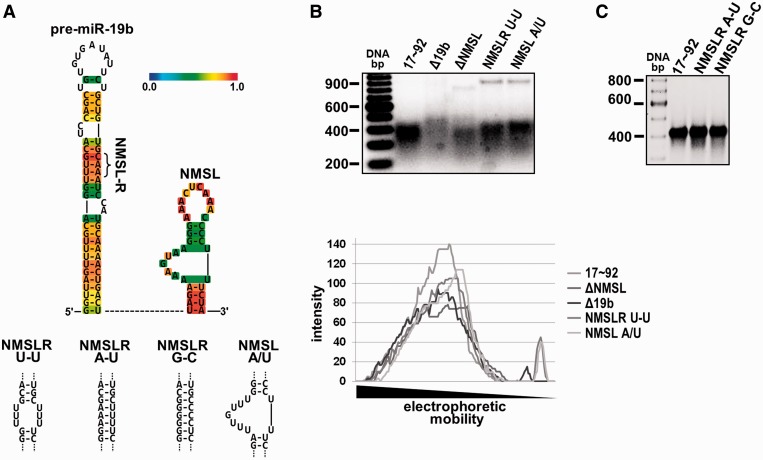
Sequence dependence of the NMSL-NMSLR interaction. (**A**) Top panel, predicted miR-19b secondary structure using CentroidHomfold (39). The color scale bar indicates base-pairing probability. Base-pairs in the stem have the highest probability. Bottom panel, miR-19b stem and NMSL mutants used in (**B**) and (**C**). (B) Top panel, deletion of the NMSL or miR-19b stem, or mutation of the adenosine repeats of the NMSL internal loop or the NMSLR yield nonhomogenous populations of folded RNAs. Bottom panel, quantification (ImageQuant) of band intensity of the native gel lanes (C) Restoring the A/U base pairing of the NMSLRU-U mutant with A/U or G/C base pairing results in homogenous populations of folded RNAs. 2.5 mM MgCl_2_ in the gel and running buffer, 5 µg RNA was loaded in each lane in both (B) and (C).

### NMSL internal loop adenosines and miR-19b stem base-pairing are required for folding miR-17∼92

Adenosine is the most frequent unpaired nucleotide in large rRNAs (19,36) and unpaired adenosines in single-stranded regions; internal loops and bulges and terminal hairpin loops frequently mediate tertiary contacts in the formation of RNA tertiary structure (29). Two adjacent base-stacked single-stranded adenosines are a defining structural feature in the A-minor motif where base-stacked single-stranded adenosines H-bond to the minor groove face of a Watson–Crick base-paired stem (28,37). The general secondary structural features of miRNA hairpins as predicted by RNA folding algorithms has been shown to be largely valid, with the only discrepancies limited to the exact base-pairing in the terminal loop region of the miRNA hairpins (38). As described above, the NMSL has a tandem adenosine repeat in the internal loop, and the NMSLR within the miR-19b hairpin contains an adenosine repeat within A-U Watson–Crick base pairs (Figure 5A) (39). Given the secondary structure of the miR-19b stem and the NMSL, it is possible that these elements are interacting via an A-minor motif (28,37). We used native gel electrophoresis to assay miR-17∼92 mutants for structure formation. Removal of the NMSL (ΔNMSL) or the miR-19b stem (Δ19b) is deleterious to forming a population of homogeneously folded RNAs resulting in multiple bands (populations) of reduced intensity compared to the single prominent wild-type band. The adenosines of the internal loop of the NMSL (mutant NMSL-A/U), or the adenosines of the NMSLR (mutant NMSLR U-U), were mutated to uridines and their effects on folding were also investigated. Either mutation alters the folding of the RNA as is evident by the presence of multiple bands of lower intensity compared with a single prominent species for the wild-type RNA (Figure 5). A qualitative quantification of the gel lanes shows the band intensities and existence of multiple species. Thus, the adenosines of the internal loop of the NMSL and the Watson–Crick base pairing in the NMSLR both appear to be important in structuring the pri-miRNA. If the interaction between the NMSL and NMSLR were through an A-minor motif, reestablishing base-pairing in the NMSLR U-U mutant should restore the interaction because the A-minor motif only requires that there be a Watson–Crick base-paired stem, with some preference for G/C base-pairing over A/U base pairing (37). To test this we compared wild-type with a rescue mutant (NMSLR A-U) that restored base-pairing in the miR-19b stem and a mutant that swopped A/U base-pairing for G/C base pairing (NMSLR G-C). Both the rescue mutant and the G/C mutant form homogenous structured RNAs comparable with the wild type as assayed by native gel electrophoresis (Figure 5C). While these results are consistent with an A-minor motif interaction between the NMSL and the NMSLR, higher resolution structural methods will be required to definitively categorize this interaction.

### NMSL-mediated tertiary structure represses miR-92a Drosha processing and mRNA targeting

**Figure 6.**
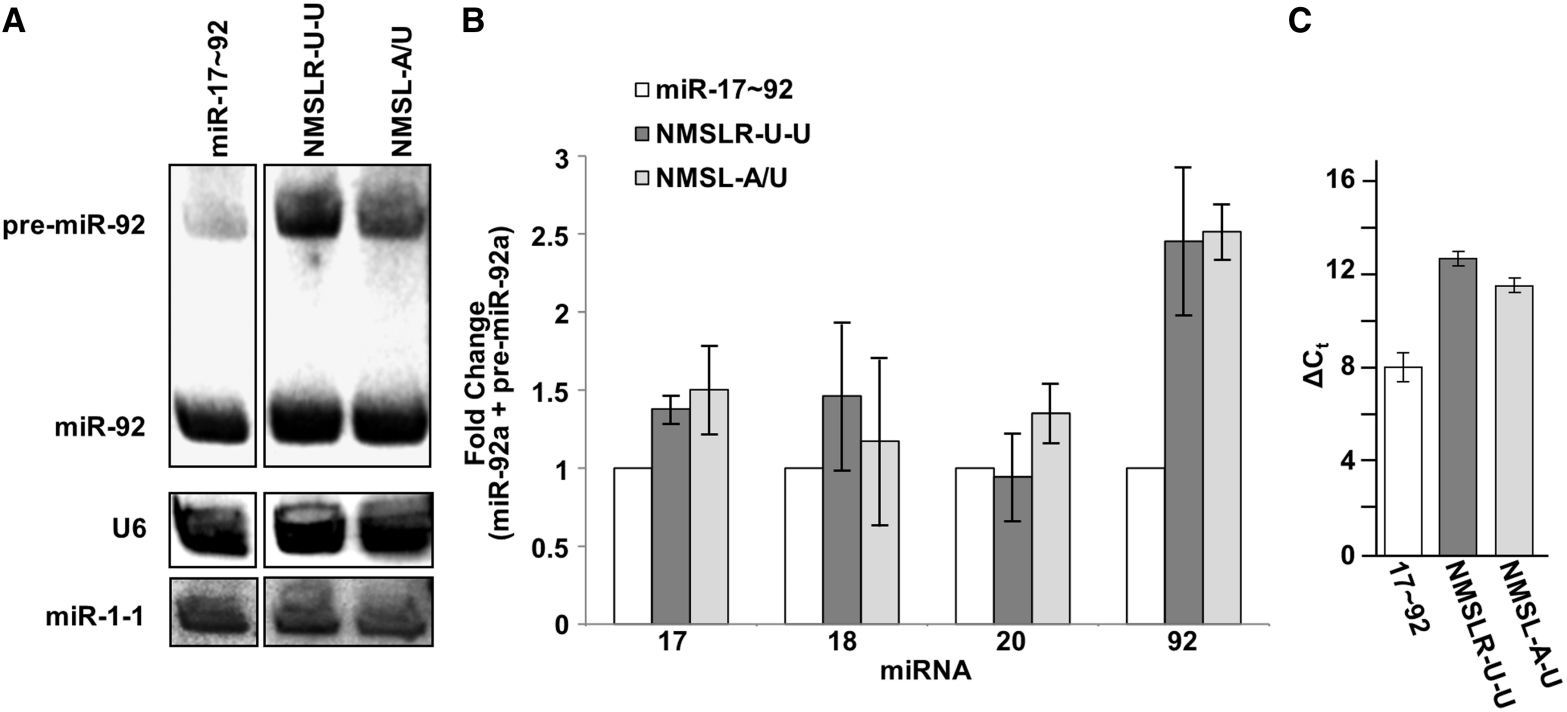
Drosha (Microprocessor) processing of miR-17∼92, NMSL-A/U and NMSLR-A/U pri-miRNA. (**A**) Northern blot of miR-92a expression from cells transiently expressing wild-type miR-17∼92, NMSLR U-U or NMSL A/U. U6 is probed as a loading control, miR-1 is a transfection efficiency control. (**B**) Total expression of the individual premature and mature miRNAs from the NMSLR-U-U and NMSL-A/U mutant clusters (corrected for background endogenous expression, lane loading and transfection efficiency), normalized to expression from the wild type pri-miRNA cluster. miR-92a expression shows the highest increase in relative expression. Data are averaged from three independent experiments. Error bars represent one standard deviation. (**C**) Comparative quantitative RT-PCR of ITGA5 mRNA from HEK293T cells expressing wild-type miR-17∼92, or the NMSLR U-U, or NMSL A/U mutants using β-actin mRNA as an internal standard generated ΔΔCt values of 4.5 ± 1.4 and 3.4 ± 1.3, respectively. Error bars indicate one standard deviation.

If the NMSL-miR-19b interaction is important for the folding of the miR-17∼92 structure, we predict that disruption of this interaction will alter the processing and maturation of some of the constituent miRNAs. We have previously demonstrated that the miR-92a hairpin is modestly processed from the pri-miR-17∼92 structure, which can be enhanced by mutations that disrupt the RNA structure (18). Drosha processing of the miR-92a hairpin from wild-type, NMSL-A/U and NMSLR-A/U mutant clusters were investigated in HEK293T cells. Northern blot analysis was performed to quantify the total amount of premature and mature miRNA in the samples. Mutations to either the NMSLR or NMSL results in increased miR-92a maturation, in comparison with wild type, while no significant change in processing was observed for the other three miRNAs (Figure 6A and B). The point mutants appear to impair the overall folding of the RNA and prevent the sequestering of the pre-miRNAs from Drosha processing. These data parallel previous data where a deletion within the 3′ core domain also results in enhanced miR-92a expression levels (18).

In addition to the direct increase in miR-92a levels, disruption of the NMSL-miR-19b interaction enhances the downregulation of a miR-92a target mRNA. We had previously established a correlation between changes in Drosha processing efficiency of miR-92a and mRNA targeting efficiency by miR-92a in cultured cells (18). We reported that disruption of cluster structure by deleting the miR-19b hairpin results in increased miR-92a levels and enhanced downregulation of ITGA5, a previously established miR-92a target (40). Similar investigations were performed with the NMSLR U-U and NMSL A/U mutants. Wild-type and mutant pri-miRNAs were transiently expressed in HEK293T cells. Quantification of ITGA5 mRNA levels by quantitative RT-PCR 48 h after transfection reveals a decrease in ITGA5 mRNA in cells expressing either mutant, relative to the wild-type cluster (Figure 6C).

## DISCUSSION

In our previous report (18) of the pri-miR-17∼92 structure we identified that the global architecture of the cluster is involved in the processing of the constituent miRNAs. Recent data from others has also revealed significant tertiary structure for the miR-17∼92 pri-miRNA (19). For a more detailed understanding of the miR-17∼92 structure we focused on identifying specific tertiary contacts involved in the folding of this large pri-miRNA into a compact globular structure. Single-stranded tandem adenosines are frequently involved in RNA tertiary structure formation, in the form of A-minor motifs, ribose zippers and tetra-loop/tetra-loop receptor interactions (29). Not surprisingly, adenosine is the most common unpaired nucleotide in large rRNA structures (36). Thus, a search for tandem single-stranded adenosines within the predicted RNA secondary structure is a simple first step in identifying sequences that are potentially mediating tertiary structure formation.

Given that the NMSL sequence is highly conserved between species containing the miR-17∼92 cluster and the presence of several (18) single-stranded adenosine repeats in the NMSL (Figure 1B), we postulated that the NMSL may be involved in mediating tertiary contacts within the miR-17∼92 structure. Supporting this hypothesis was the internalization of the NMSL within the 3′ core domain itself (Figure 2), it being required in folding the full-length cluster (18) and the presence of single-stranded base-stacked adenosines (Figure 3).

A zero length cross-linker was used to identify potential tertiary contacts within the RNA. The cross-linker 4SU (41) is a photoactive base that we used in RNA cross-linking experiments (30) to identify potential tertiary contact with the adenosine repeats of the NMSL loops. The single substitution of oxygen with sulfur provides a photoactive base without bulky photoactive functional groups, like benzophenone (42), which can create steric clashes in closely packed RNA structures. Site-specific incorporation of 4SU in the 3′ core domain was accomplished using a splinted ligation procedure (33). The formation of ultraviolet-dependent cross-linked lariats, when the 4SU was at position 685, indicates that the NMSL forms tertiary contacts from the internal loop to regions outside of the NMSL (Figure 4A). The cross-link was mapped to a double-stranded region within the miR-19b hairpin stem, which we have termed the NMSLR (Figure 4B and C). The requirement of single-stranded base-stacked adenosines in the NMSL and Watson–Crick base-pairing within the NMSLR (Figure 5), is consistent with a hypothesis that the NMSL-NMSLR tertiary interaction may be mediated by base triple formation in an A-minor motif, a prevalent structural motif in structured RNAs (37,43).

Disruption of the folding of the pri-miR-17∼92 structure alters the differential expression of the constituent miRNAs. We have previously reported that tertiary structure of the pri-miR-17∼92 cluster suppresses the processing of the 3′ core domain miRNAs miR-19b and miR-92a as well as miR-18a. Though not part of the core domain, miR-18a is also internalized within the globular cluster structure. (Figure 1) (18). In these investigations, disruption of the pri-miRNA structure, by removal of the miR-19b miRNA hairpin from the 3′ core domain, resulted in increased miR-92a levels and mRNA targeting in HEK29T cells. To a lesser extent, removal of miR-19b also resulted in increased miR-18a expression, suggesting miR-18a is involved in tertiary interactions outside the 3′ core domain. We have now demonstrated that the NMSL is involved in a tertiary contact with the miR-19b hairpin through the NMSLR (Figure 4) and the removal of this small stem-loop (Figure 5) or mutations to either the NMSL or NMSLR (Figure 5) disrupts the structure of the pri-miRNA cluster. These mutations to the NMSL or NMSLR enhance miR-92a expression (Figure 6A) as was previously described with the miR-19b hairpin deletion (18). Similarly, the expression of the mutant pri-miR-17∼92 clusters NMSLR U-U or NMSL A/U results in enhanced downregulation of ITGA5 mRNA (Figure 6C), a previously reported miR-92a target (40).

Reduced expression of miR-92a from the pri-miR-17∼92 cluster as a result of the pri-miRNA structure fits with the reported antagonistic role of this miRNA (44). Through the combined action of several constituent miRNAs, miR-17∼92 expression has been shown to facilitate tumor angiogenesis by repression of antiangiogenic proteins Tsp1 and CTGF by miR-18a and miR-19a, (13) miR-17 and miR-20 targeting of the TGFβ tumor suppressor pathway (45,46) and miR-17 and miR-20 targeting the E2F transcription factor family (47,48). These effects are in contrast to the reported antiangiogenic activity of miR-92a (40). Thus miR-92a, a 3′ core miRNA, appears to have an antagonistic effect with respect to expression of the other miRNAs originating from this cluster. We have shown that structure within the miR-17∼92 pri-miRNA minimizes miR-92a expression. The NMSL-NMSLR tertiary interaction is a key tertiary structure element involved in repression of miR-92a by stabilizing the miR-17∼92 structure. We propose that in this manner, the NMSL–NMSLR tertiary interaction suppresses miR-92a expression, allowing the net pro-angiogenic biological effect of miR-17∼92 expression. The low level of miR-92a expression, relative to noncore miRNAs, on c-Myc induction of miR-17∼92 expression (49) supports this model. It has been shown that hnRNNPA1, through binding the miR-18a terminal loop, enhances miR-18a expression (50). As well, miR-92a is frequently found to be highly expressed in several tissues and tumor types (51). The next key step in the investigation of RNA-structure–based modulation of miRNA expression is to identify additional RNA binding cofactors that may alter the pri-miRNA structure to unlock the structured-based repression of miR-92a expression relative to the other constituent miRNAs of miR-17∼92.

## SUPPLEMENTARY DATA

Supplementary Data are available at NAR Online.

## FUNDING

National Cancer Institute (NCI to M.J.N.G.); Canadian Breast Cancer Foundation (CBCF) Prairies/NWT region (to R.P.F.). Funding for open access charge: Canadian Breast Cancer Foundation.

*Conflict of interest statement*. None declared.

## Supplementary Material

Supplementary Data

## References

[1] Bartel DP (2004). MicroRNAs: genomics, biogenesis, mechanism, and function. Cell.

[2] Cabibihan JJ (2011). Patient-specific prosthetic fingers by remote collaboration–a case study. PLoS One.

[3] Olive V, Jiang I, He L mir-17-92, a cluster of miRNAs in the midst of the cancer network. Int. J. Biochem. Cell Biol..

[4] Ohtani K, Dimmeler S Control of cardiovascular differentiation by microRNAs. Basic Res. Cardiol..

[5] Mendell JT (2008). miRiad roles for the miR-17-92 cluster in development and disease. Cell.

[6] Osada H, Takahashi T let-7 and miR-17-92: small-sized major players in lung cancer development. Cancer Sci..

[7] Kim VN (2005). MicroRNA biogenesis: coordinated cropping and dicing. Nat. Rev. Mol. Cell Biol..

[8] Russo G, Giordano A (2009). miRNAs: from biogenesis to networks. Methods Mol. Biol..

[9] Filipowicz W, Bhattacharyya SN, Sonenberg N (2008). Mechanisms of post-transcriptional regulation by microRNAs: are the answers in sight?. Nat. Rev. Genet..

[10] Garzon R, Calin GA, Croce CM (2009). MicroRNAs in cancer. Annu. Rev. Med..

[11] Esquela-Kerscher A, Slack FJ (2006). Oncomirs - microRNAs with a role in cancer. Nat. Rev. Cancer.

[12] He L, Thomson JM, Hemann MT, Hernando-Monge E, Mu D, Goodson S, Powers S, Cordon-Cardo C, Lowe SW, Hannon GJ (2005). A microRNA polycistron as a potential human oncogene. Nature.

[13] Dews M, Homayouni A, Yu D, Murphy D, Sevignani C, Wentzel E, Furth EE, Lee WM, Enders GH, Mendell JT (2006). Augmentation of tumor angiogenesis by a Myc-activated microRNA cluster. Nat. Genet..

[14] Ventura A, Young AG, Winslow MM, Lintault L, Meissner A, Erkeland SJ, Newman J, Bronson RT, Crowley D, Stone JR (2008). Targeted deletion reveals essential and overlapping functions of the miR-17 through 92 family of miRNA clusters. Cell.

[15] Han J, Lee Y, Yeom KH, Nam JW, Heo I, Rhee JK, Sohn SY, Cho Y, Zhang BT, Kim VN (2006). Molecular basis for the recognition of primary microRNAs by the Drosha-DGCR8 complex. Cell.

[16] Zeng Y, Cullen BR (2005). Efficient processing of primary microRNA hairpins by Drosha requires flanking nonstructured RNA sequences. J. Biol. Chem..

[17] Auyeung VC, Ulitsky I, McGeary SE, Bartel DP (2013). Beyond secondary structure: primary-sequence determinants license pri-miRNA hairpins for processing. Cell.

[18] Chaulk SG, Thede GL, Kent OA, Xu Z, Gesner E, Veldhoen RA, Khanna SK, Goping IS, Macmillan AM, Mendell JT (2011). Role of pri-miRNA tertiary structure in miR-17∼92 miRNA biogenesis. RNA Biol..

[19] Chakraborty S, Mehtab S, Patwardhan A, Krishnan Y (2012). Pri-miR-17-92a transcript folds into a tertiary structure and autoregulates its processing. RNA.

[20] Graham FL, van der Eb AJ (1973). A new technique for the assay of infectivity of human adenovirus 5 DNA. Virology.

[21] Meador J, Cannon B, Cannistraro VJ, Kennell D (1990). Purification and characterization of *Escherichia coli* RNase I. Comparisons with RNase M. Eur. J. Biochem..

[22] Lowman HB, Draper DE (1986). On the recognition of helical RNA by cobra venom V1 nuclease. J. Biol. Chem..

[23] Arthur DC, Edwards RA, Tsutakawa S, Tainer JA, Frost LS, Glover JN (2011). Mapping interactions between the RNA chaperone FinO and its RNA targets. Nucleic Acids Res..

[24] Serebrov V, Clarke RJ, Gross HJ, Kisselev L (2001). Mg2+-induced tRNA folding. Biochemistry.

[25] Zuker M (2003). Mfold web server for nucleic acid folding and hybridization prediction. Nucleic Acids Res..

[26] Bernal JJ, Garcia-Arenal F (1997). Analysis of the in vitro secondary structure of cucumber mosaic virus satellite RNA. RNA.

[27] Lowman HB, Draper DE (1986). On the recognition of helical RNA by cobra venom V1 nuclease. J. Biol. Chem..

[28] Doherty EA, Batey RT, Masquida B, Doudna JA (2001). A universal mode of helix packing in RNA. Nat. Struct. Biol..

[29] Xin Y, Laing C, Leontis NB, Schlick T (2008). Annotation of tertiary interactions in RNA structures reveals variations and correlations. RNA.

[30] Hiley SL, Sood VD, Fan J, Collins RA (2002). 4-thio-U cross-linking identifies the active site of the VS ribozyme. EMBO J..

[31] Bravo C, Lescure F, Laugaa P, Fourrey JL, Favre A (1996). Folding of the HDV antigenomic ribozyme pseudoknot structure deduced from long-range photocrosslinks. Nucleic Acids Res..

[32] Pinard R, Heckman JE, Burke JM (1999). Alignment of the two domains of the hairpin ribozyme-substrate complex defined by interdomain photoaffinity crosslinking. J. Mol. Biol..

[33] Moore MJ, Query CC (2000). Joining of RNAs by splinted ligation. Methods Enzymol..

[34] Laugaa P, Woisard A, Fourrey JL, Favre A (1995). Hammerhead ribozyme: a three dimensional model based on photo-crosslinking data. C. R. Acad. Sci. III.

[35] Juzumiene DI, Wollenzien P (2001). Arrangement of the central pseudoknot region of 16S rRNA in the 30S ribosomal subunit determined by site-directed 4-thiouridine crosslinking. RNA.

[36] Nissen P, Ippolito JA, Ban N, Moore PB, Steitz TA (2001). RNA tertiary interactions in the large ribosomal subunit: the A-minor motif. Proc. Natl Acad. Sci. USA.

[37] Battle DJ, Doudna JA (2002). Specificity of RNA-RNA helix recognition. Proc. Natl Acad. Sci. USA.

[38] Krol J, Sobczak K, Wilczynska U, Drath M, Jasinska A, Kaczynska D, Krzyzosiak WJ (2004). Structural features of microRNA (miRNA) precursors and their relevance to miRNA biogenesis and small interfering RNA/short hairpin RNA design. J. Biol. Chem..

[39] Hamada M, Yamada K, Sato K, Frith MC, Asai K (2011). CentroidHomfold-LAST: accurate prediction of RNA secondary structure using automatically collected homologous sequences. Nucleic Acids Res..

[40] Bonauer A, Carmona G, Iwasaki M, Mione M, Koyanagi M, Fischer A, Burchfield J, Fox H, Doebele C, Ohtani K (2009). MicroRNA-92a controls angiogenesis and functional recovery of ischemic tissues in mice. Science.

[41] Juzumiene D, Shapkina T, Kirillov S, Wollenzien P (2001). Short-range RNA-RNA crosslinking methods to determine rRNA structure and interactions. Methods.

[42] Konarska MM (1999). Site-specific derivatization of RNA with photocrosslinkable groups. Methods.

[43] Batey RT, Rambo RP, Doudna JA (1999). Tertiary motifs in RNA structure and folding. Angew. Chem. Int. Ed. Engl..

[44] Li Y, Vecchiarelli-Federico LM, Li YJ, Egan SE, Spaner D, Hough MR, Ben-David Y (2012). The miR-17-92 cluster expands multipotent hematopoietic progenitors whereas imbalanced expression of its individual oncogenic miRNAs promotes leukemia in mice. Blood.

[45] Petrocca F, Vecchione A, Croce CM (2008). Emerging role of miR-106b-25/miR-17-92 clusters in the control of transforming growth factor beta signaling. Cancer Res..

[46] Dews M, Fox JL, Hultine S, Sundaram P, Wang W, Liu YY, Furth E, Enders GH, El-Deiry W, Schelter JM (2010). The myc-miR-17∼92 axis blunts TGF{beta} signaling and production of multiple TGF{beta}-dependent antiangiogenic factors. Cancer Res..

[47] Woods K, Thomson JM, Hammond SM (2007). Direct regulation of an oncogenic micro-RNA cluster by E2F transcription factors. J. Biol. Chem..

[48] Pickering MT, Stadler BM, Kowalik TF (2009). miR-17 and miR-20a temper an E2F1-induced G1 checkpoint to regulate cell cycle progression. Oncogene.

[49] O'Donnell KA, Wentzel EA, Zeller KI, Dang CV, Mendell JT (2005). c-Myc-regulated microRNAs modulate E2F1 expression. Nature.

[50] Guil S, Caceres JF (2007). The multifunctional RNA-binding protein hnRNP A1 is required for processing of miR-18a. Nat. Struct. Mol. Biol..

[51] Landgraf P, Rusu M, Sheridan R, Sewer A, Iovino N, Aravin A, Pfeffer S, Rice A, Kamphorst AO, Landthaler M (2007). A mammalian microRNA expression atlas based on small RNA library sequencing. Cell.

