# Supplementary material for: MicroRNA miR-92a-1 biogenesis and mRNA targeting is modulated by a tertiary contact within the miR-17∼92 microRNA cluster
Source: Nucleic Acids Res. 2014 Feb 11;42(8):5234–44. doi: 10.1093/nar/gku133 (PMC4005684; doi:10.1093/nar/gku133)
Supplement: Supplementary Data [file supp_42_8_5234__index.html]

MicroRNA miR-92a-1 biogenesis and mRNA targeting is modulated by a tertiary contact within the miR-17∼92 microRNA cluster — Supplementary Data 

# MicroRNA miR-92a-1 biogenesis and mRNA targeting is modulated by a tertiary contact within the miR-17∼92 microRNA cluster

## Supplementary Data

files

**Files in this Data Supplement:**

- Supplementary Data - pdf file
