# Supplementary material for: MicroRNA miR-92a-1 biogenesis and mRNA targeting is modulated by a tertiary contact within the miR-17∼92 microRNA cluster
Source: Nucleic Acids Res. 2014 Feb 11;42(8):5234–44. doi: 10.1093/nar/gku133 (PMC4005684; doi:10.1093/nar/gku133)
Supplement: Supplementary Data [file supp_gku133_nar-03289-x-2013-File008.pdf]

Supplementary Data

MicroRNA miR-92a-1 Biogenesis and mRNA Targeting is Modulated by a Tertiary Contact within the miR-17~92 microRNA cluster

Chaulk, S.G.[1,*], Zhizhong Xu[2], Glover, J.N.[1], Richard P. Fahlman[1,2,*]

[1]Department of Biochemistry, [2]Department of Oncology, University of Alberta, Edmonton, Alberta, Canada, T6G 2H7.

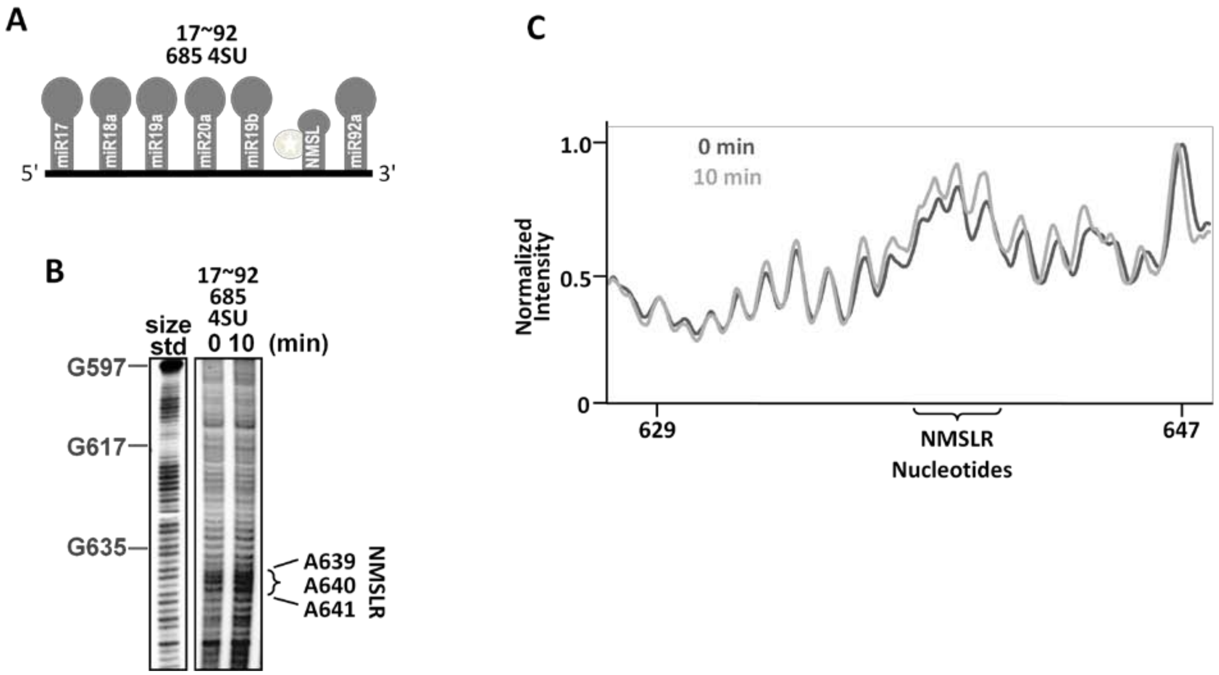

Supplemental Figure 1. Tertiary contact between the NMSL and NMSLR. (A) Schematic of 4SU incorporation into the 17~92 cluster 3' for photo-cross-linking. (B) Cross-link dependent reverse transcription stops indicate the positions of the nucleotides cross-linked to the 4SU within 17~92. Positions of size standards corresponding to positions G597, G617 and G635 are indicated on a DNA ladder. Significant increases in band intensity are only observed for the NMSLR region. The 685 4SU 17~92 RNA was prepared as described in the materials and methods section. The 5' and 3' portions for ligation to the 4-thio-U RNA were transcribed from PCR generated T7 RNA polymerase templates.
